# From Cellulose to Nanocellulose: Functionalization Strategies and Applications in Biomedicine, Ecology, and Energy

**DOI:** 10.3390/polym18111300

**Published:** 2026-05-25

**Authors:** Akmaral Darmenbayeva, Reshmy Rajasekharan, Bakytgul Massalimova, Murshida Aimova, Nurbala Ubaidulayeva, Gulzhan Abylkassova, Shynar Sanyazova, Rekha Unni, Dinislam Khuzin, Musrepbek Kurmanaliev, Zhazira Mukazhanova

**Affiliations:** 1 Department of Chemistry and Chemical Technology, M.Kh. Dulaty Taraz University, Taraz 080000, Kazakhstan; 2 Department of Science and Humanities, Providence College of Engineering, Thiruvananthapuram 689122, Kerala, India; reshmypkumar@gmail.com; 3 Department of Chemistry and Chemical Technology, M. Kozybayev North Kazakhstan University, Petropavlovsk 150000, Kazakhstan; bkmasalimova@ku.edu.kz; 4 Department of Natural Sciences, Yessenov University, Aktau 130000, Kazakhstan; murshida.aimova@yu.edu.kz; 5 Higher School of Natural Sciences, Margulan University, Pavlodar 140000, Kazakhstan; nurbala-76@mail.ru; 6 Department of Chemistry, S. Amanzholov East Kazakhstan University, Ust-Kamenogorsk 070010, Kazakhstan; abylkassova@mail.ru (G.A.); shynarsanyazova@mail.ru (S.S.); 7 Department of Chemistry, Christian College Chengannur, University of Kerala, Thiruvananthapuram 689122, Kerala, India; rekhaunni08@gmail.com; 8 Department of General Chemistry, Bashkir State Medical University, Ufa 450000, Russia; dinislamkhuzin@mail.ru; 9 Department of Chemistry and Chemical Technology, Almaty Technological University, Almaty 050002, Kazakhstan; mkk@mail.ru

**Keywords:** cellulose functionalization, nanocellulose, surface modification, biomedical applications, energy and environmental technologies

## Abstract

The growing demand for sustainable and high-performance materials has positioned cellulose as a key biopolymer for next-generation functional systems. Beyond its traditional use, cellulose undergoes a qualitative transformation at the nanoscale, where increased surface area, interfacial dominance, and tunable chemistry enable functions unattainable in bulk form. This review provides a critical and integrative analysis of functionalization strategies governing the transition from structural modification to application-specific performance in cellulose and nanocellulose-based materials. A unified structure–property–function–process (SPFP) framework is introduced to systematically connect modification approaches with resulting structural features, physicochemical properties, and functional outcomes. Chemical, physical, and surface/interface modification strategies are comparatively evaluated with respect to their efficiency, scalability, and environmental trade-offs. Rather than cataloguing methods, the review emphasizes cross-domain synthesis and identifies key limitations, including high energy demand, reagent consumption, structural instability, and challenges in large-scale implementation. Particular attention is given to applications in biomedicine, environmental remediation, and energy technologies, where performance is governed by surface reactivity, accessibility, and hierarchical organization. The analysis highlights that no single modification strategy is universally optimal, and that effective material design requires balancing performance, sustainability, and process feasibility. By integrating conceptual frameworks, comparative analysis, and emerging design principles, this review provides a forward-looking perspective on the development of cellulose-based functional materials, supporting their transition from laboratory-scale demonstrations to application-ready technologies.

## 1. Introduction

Cellulose is the most abundant natural polysaccharide and an essential structural component of the plant cell wall. Its unique combination of biodegradability, renewability, biocompatibility, and high mechanical strength has made cellulose one of the most studied biopolymers for sustainable materials development [1,2,3]. For decades, it has found wide application in paper, textile, and food industries, as well as in pharmaceuticals and medicine. However, native cellulose has inherent limitations, including poor solubility in most solvents, limited chemical reactivity, and restricted functionality, which reduce its ability to meet the demands of advanced technologies [4].

In this review, chemical and physical modification strategies are considered as enabling steps that lead to cellulose functionalization, understood here as the acquisition of application-specific properties [5,6]. These methods improve cellulose performance by enhancing solubility, adsorption capacity, and conductivity. As a result, functionalized cellulose has been increasingly investigated as a platform material for biomedical, environmental, and energy-related applications.

A particularly significant advance in the last two decades is the emergence of nanocellulose, including cellulose nanocrystals (CNCs), cellulose nanofibrils (CNFs), and bacterial cellulose (BC). Rather than focusing on a single nanocellulose type, this review comparatively examines CNCs, CNFs, and bacterial nanocellulose because their distinct morphologies, crystallinity, surface accessibility, and interfacial behavior lead to fundamentally different functionalization pathways and application performance. Such comparative analysis is essential for establishing generalized structure–property–function relationships and for identifying the advantages and limitations of different nanocellulose platforms within the proposed SPFP framework. Nanocellulose exhibits exceptionally high surface area, tunable surface chemistry, low density, and superior mechanical properties, making it suitable for modern applications such as drug delivery, biosensing, pollutant removal, flexible electronics, and sustainable energy storage devices [7,8]. Recent reviews emphasize that functionalized nanocellulose represents a key step toward replacing petroleum-based polymers with sustainable alternatives [9,10].

Recent studies published in 2024–2025 further strengthen this trend by demonstrating how nanocellulose structure and surface chemistry govern functional performance in biomedical and pharmaceutical systems. Al-Zu’bi et al. highlighted diverse production methods and functionalization strategies of nanocellulose for advanced medical and pharmaceutical applications, focusing on membranes, hydrogels, and drug delivery systems [11]. Rafee et al. provided a systematic overview of nanocellulose polymorphs (CNC, CNF, BC) and their biomedical potential, underscoring how structural variety determines functional performance [12]. Meanwhile, Thomas et al. emphasized sustainable sources by extracting and characterizing nanocellulose from date palm biomass, demonstrating the prospects of agro-industrial waste as a feedstock for high-value nanomaterials [13].

Despite significant progress, the literature on cellulose functionalization remains fragmented, with many studies focusing either on bulk cellulose derivatives or on nanocellulose-based systems, often without a unified perspective. To address this gap, the present review systematically examines modification strategies enabling cellulose functionalization and critically analyzes the resulting functionalized cellulose and nanocellulose materials in the contexts of biomedicine, ecology, and energy.

The central premise of this review extends beyond the conventional description of functionalization strategies. Despite significant progress in cellulose and nanocellulose research, the translation of functionalized systems into practical technologies remains challenging. This limitation is mainly associated with trade-offs between performance, scalability, sustainability, and long-term stability. While many studies report enhanced adsorption capacity, biocompatibility, or conductivity, these improvements are often accompanied by increased process complexity, higher chemical consumption, limited recyclability, or insufficient durability under realistic operating conditions.

Therefore, this review adopts a critical perspective, applying a structure–property–function framework to evaluate how specific modification strategies influence material performance and where they fail to meet application requirements. Particular attention is given to conflicting findings, performance limitations, and unresolved challenges that are frequently overlooked in descriptive reviews. By identifying key knowledge gaps and practical constraints, this work aims to move beyond cataloguing existing approaches toward a more analytical and forward-looking understanding of functionalized cellulose systems. Unlike many existing reviews that primarily summarize modification methods or focus on a single application domain, the present review introduces a unified structure–property–function–process (SPFP) framework that comparatively evaluates chemical, physical, and surface/interface functionalization strategies across biomedical, environmental, and energy-related systems. Particular emphasis is placed on identifying trade-offs between functional performance, scalability, regeneration stability, environmental impact, and long-term operational durability. Thus, the advancement of this review lies in its cross-domain critical analysis and integrative perspective linking modification pathways with practical implementation challenges and application-specific performance.

To ensure conceptual clarity, the terms “modification”, “functionalization”, and “engineering” are used with distinct meanings throughout this review. “Modification” refers to any chemical, physical, or surface alteration of cellulose structure. “Functionalization” denotes the introduction of targeted functionalities that enable specific application-driven performance. “Engineering” is used in a broader sense to describe the rational design of cellulose-based systems, integrating modification strategies with structure–property–function relationships and processing constraints.

## 2. Methodology of Literature Selection and Analysis

This review is based on a structured analysis of recent literature on cellulose and nanocellulose functionalization, with a particular focus on structure–property–function relationships and application performance.

Scientific publications were retrieved from major databases including Scopus, Web of Science, and ScienceDirect. The search was conducted using combinations of keywords such as “cellulose functionalization”, “nanocellulose”, “surface modification”, “cellulose-based materials”, “adsorption”, “biomedical applications”, and “energy applications”.

Priority was given to peer-reviewed articles published between 2020 and 2025 to ensure coverage of recent advances. Earlier studies were included selectively to provide foundational context. The selection criteria focused on studies reporting clear relationships between modification strategies, physicochemical properties, and functional performance.

Rather than following a strictly systematic review protocol, this work adopts a critical comparative approach. The selected studies were analyzed in terms of modification efficiency, performance metrics, scalability, and environmental impact. Particular attention was given to identifying limitations, conflicting results, and gaps in current research.

This approach enables a balanced and reproducible evaluation of the field while maintaining the flexibility required for a critical review.

## 3. From Cellulose to Nanocellulose: Structural and Chemical Transformation

### 3.1. Cellulose Sources Relevant to Nanocellulose Production

The choice of cellulose source is a critical factor determining the structural organization, crystallinity, surface chemistry, and functionalization potential of nanocellulose. Although cellulose is widely available from diverse biological origins, only selected feedstocks enable the reproducible production of cellulose nanocrystals (CNCs), cellulose nanofibrils (CNFs), and bacterial nanocellulose (BNC) with controlled morphology and surface reactivity suitable for advanced functional applications [14,15,16]. Understanding source-dependent features is therefore essential for rational material design in biomedical, environmental, and energy-related fields.

Plant-derived cellulose remains the predominant source for nanocellulose production. This is mainly due to its abundance, renewability, and well-established industrial infrastructure. Wood pulp and non-wood plant fibers possess a hierarchical organization in which semicrystalline cellulose microfibrils are embedded within amorphous regions. This structural anisotropy enables efficient conversion into CNCs and CNFs with high aspect ratios, tunable crystallinity, and a high density of surface hydroxyl groups [14,17]. These characteristics are particularly advantageous for chemical modification strategies such as oxidation, esterification, and etherification, which rely on accessible hydroxyl functionalities. Extensive studies have demonstrated that plant-based nanocellulose exhibits excellent mechanical reinforcement capability, favorable interfacial interactions, and chemical versatility, making it a robust platform for functionalized composites, membranes, and biomedical materials [15,18].

Beyond conventional wood-based feedstocks, agro-industrial residues have emerged as promising alternative sources of cellulose for nanocellulose production. Agricultural by-products and processing wastes represent renewable and low-cost resources that align with circular bioeconomy principles. Recent studies indicate that nanocellulose derived from agro-industrial residues can exhibit physicochemical properties comparable to those obtained from virgin wood pulp, provided appropriate pretreatment and fibrillation strategies are employed [19,20]. Importantly, the primary advantage of such sources lies not in maximizing cellulose yield but in enhancing sustainability, scalability, and environmental performance. Nanocellulose produced from agro-industrial residues has been successfully explored for functional applications including pollutant adsorption, filtration membranes, and hybrid composite systems, highlighting its potential for large-scale deployment in environmental technologies [21].

Bacterial cellulose represents a distinct and highly attractive class of nanocellulose produced directly in nanoscale form through microbial biosynthesis. Unlike plant-derived cellulose, bacterial cellulose is free from lignin, hemicelluloses, and other biogenic impurities, resulting in an ultrafine three-dimensional nanofibrillar network with exceptional purity, high porosity, and superior water-holding capacity [22]. This intrinsic nanostructure provides a highly reactive and uniform surface that is particularly well suited for chemical and physical functionalization without extensive pretreatment. Due to its excellent biocompatibility, mechanical robustness, and structural stability, bacterial cellulose has been extensively investigated for biomedical applications, including wound dressings, tissue engineering scaffolds, and implantable devices [23,24]. Moreover, its well-defined nanoscale architecture enables precise control over surface modification, which is critical for tailoring biological responses and interfacial properties.

The main cellulose sources used for nanocellulose production, and their key structural and functional characteristics, are summarized in Table 1.

Collectively, plant-derived cellulose, agro-industrial residues, and bacterial cellulose constitute complementary feedstocks for nanocellulose production. Each source offers distinct structural and chemical characteristics that directly influence nanocellulose morphology, surface reactivity, and functionalization efficiency. Recognizing these source-dependent features provides a necessary foundation for selecting appropriate modification strategies and for developing functionalized nanocellulose materials with application-specific performance, as discussed in the subsequent sections of this review.

### 3.2. Types of Nanocellulose and Key Physicochemical Properties

Nanocellulose encompasses a family of cellulose-based nanomaterials distinguished by their morphology, dimensions, crystallinity, and surface chemistry. The three principal types―cellulose nanocrystals (CNCs), cellulose nanofibrils (CNFs), and bacterial nanocellulose (BNC)―exhibit markedly different physicochemical properties, which directly determine their functionalization behavior and suitability for specific biomedical, environmental, and energy-related applications. Understanding these differences is essential for selecting appropriate modification strategies and for tailoring nanocellulose performance. In addition to CNCs and CNFs derived from plant cellulose, bacterial nanocellulose (BNC) represents a distinct nanostructured category characterized by its intrinsically nanoscale fibrillar architecture.

Cellulose nanocrystals are rod-like nanoparticles typically obtained through controlled acid hydrolysis of cellulose, resulting in the selective removal of amorphous domains. CNCs are characterized by high crystallinity, narrow size distributions, and well-defined anisotropic geometry, with lengths ranging from tens to several hundreds of nanometers and diameters of a few nanometers. Their highly ordered crystalline structure gives CNCs exceptional stiffness and mechanical strength. As a result, CNCs are attractive reinforcing agents for nanocomposites and functional coatings [25]. From a functionalization perspective, CNCs possess a high density of surface hydroxyl groups, and depending on the hydrolysis route, may also carry charged functionalities that enhance colloidal stability. These surface features facilitate further chemical modification, enabling the introduction of bioactive, catalytic, or conductive moieties. Comprehensive analyses emphasize that the rigidity and surface regularity of CNCs are particularly advantageous for applications requiring controlled surface interactions, such as biosensing, drug delivery, and optoelectronic systems [26].

Cellulose nanofibrils consist of long, flexible fibrillar structures produced by mechanical or chemo-mechanical disintegration of cellulose fibers. Unlike CNCs, CNFs contain both crystalline and amorphous regions, resulting in a network-like morphology with high aspect ratios and extensive entanglement. This structural flexibility leads to outstanding film-forming ability, high toughness, and significant water retention capacity [27]. The large specific surface area and abundance of accessible hydroxyl groups render CNFs highly responsive to surface functionalization and interfacial engineering. Studies have demonstrated that CNFs can be readily functionalized to tailor surface charge, hydrophilicity, and interaction with polymers or biomolecules, making them particularly suitable for hydrogel systems, filtration membranes, and flexible substrates [28]. Importantly, the hierarchical fibrillar architecture of CNFs enables multifunctional behavior, where mechanical reinforcement, mass transport, and surface activity can be simultaneously optimized.

Bacterial nanocellulose is synthesized extracellularly by specific microorganisms, resulting in a three-dimensional nanofibrillar network formed directly at the nanoscale. In contrast to plant-derived nanocellulose, BNC exhibits exceptionally high purity, uniform fibril dimensions, and a highly porous structure with remarkable water-holding capacity. These intrinsic features impart excellent mechanical integrity, dimensional stability, and biocompatibility [29]. The nanoscale architecture of BNC provides a unique platform for surface functionalization, as chemical or physical modifications can be introduced without disrupting the underlying network. This makes BNC particularly attractive for biomedical applications, where precise control over surface chemistry and biological interactions is critical. Recent reviews highlight that the combination of native nanostructure and functionalization versatility enables BNC-based materials to support cell growth, controlled drug release, and bioelectronic interfaces [30]. Although bacterial nanocellulose is most commonly utilized in its native three-dimensional nanofibrillar network form, recent studies suggest that it can also be processed into nanocrystal-like or fragmented nanostructures through controlled mechanical, chemical, or enzymatic treatments. However, compared to plant-derived cellulose, the production of BNC-derived nanocrystals remains less developed, with challenges related to yield, structural control, and scalability. As a result, BNC is more appropriately considered a distinct nanocellulose platform characterized by its inherent nanoscale architecture, rather than a direct precursor to conventional CNCs or CNFs.

Although CNCs, CNFs, and BNC share a common chemical backbone, their distinct morphologies and physicochemical properties lead to fundamentally different functionalization pathways and application profiles. CNCs are best suited for applications requiring rigidity, dimensional control, and anisotropic reinforcement, whereas CNFs excel in systems demanding flexibility, permeability, and network formation. BNC occupies a unique position due to its intrinsic nanostructure and purity, offering unparalleled opportunities for biomedical and interfacial functionalization. These differences underscore the importance of matching nanocellulose type with targeted functionalization strategies, as discussed in the following section.

A comparative overview of the key physicochemical properties of CNCs, CNFs, and BNC is provided in Table 2.

**Figure 1:**
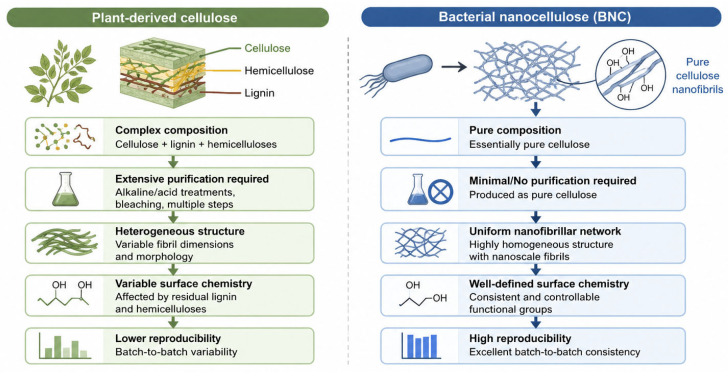
Schematic comparison of plant-derived cellulose and bacterial nanocellulose (BNC) highlighting differences in composition, purification requirements, structural uniformity, and their implications for functionalization efficiency and application performance.

The compositional and structural differences between plant-derived cellulose and bacterial nanocellulose are illustrated schematically in Figure 1. These differences have direct implications for purification, functionalization efficiency, and reproducibility, particularly in applications requiring precise control of surface chemistry. The compositional and structural differences between plant-derived cellulose and bacterial nanocellulose are illustrated schematically in Figure 1, emphasizing how purity, fibrillar organization, and structural uniformity influence purification requirements, surface accessibility, and subsequent functionalization efficiency.

Although CNCs, CNFs, and BNC share a common chemical backbone, their distinct morphologies and physicochemical properties lead to fundamentally different functionalization pathways and application profiles.

**Figure 2:**
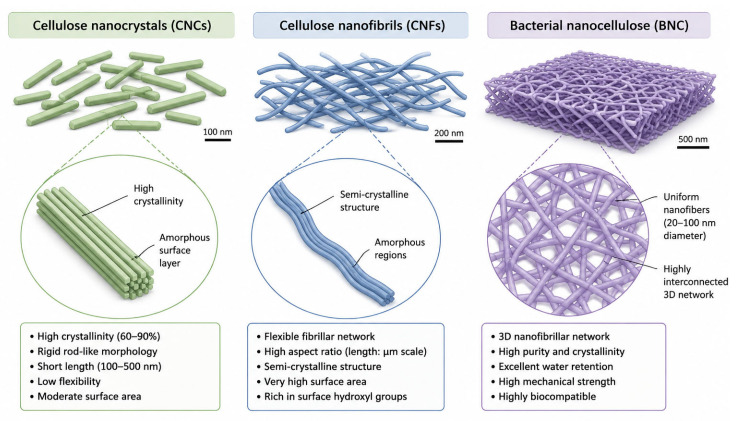
Schematic comparison of the morphology and structural organization of cellulose nanocrystals (CNCs), cellulose nanofibrils (CNFs), and bacterial nanocellulose (BNC).

The characteristic structural organization and morphology of the major nanocellulose types are schematically illustrated in Figure 2. The comparison highlights differences in fibrillar arrangement, crystallinity, and nanoscale architecture, which directly influence surface accessibility, interfacial interactions, and functionalization efficiency.

### 3.3. Rationale for Functionalization of Nanocellulose Beyond Bulk Cellulose

The transition from bulk cellulose to nanocellulose fundamentally alters not only the size and morphology of the material but also its physicochemical behavior and interaction mechanisms. While native cellulose has long been used in traditional applications, its nanoscale derivatives exhibit emergent properties that necessitate tailored functionalization strategies to fully exploit their potential in advanced technologies.

One of the most significant distinctions between bulk cellulose and nanocellulose lies in the dramatic increase in specific surface area upon nanoscale fibrillation or crystallization. Nanocellulose materials expose a substantially higher fraction of surface hydroxyl groups, leading to enhanced surface energy and increased susceptibility to interfacial interactions. This high surface area amplifies both desirable effects, such as improved adsorption capacity and interfacial bonding, and undesirable effects, including aggregation and poor dispersion stability. Consequently, functionalization becomes essential to regulate surface interactions, prevent uncontrolled agglomeration, and ensure stable performance in colloidal systems, composites, and membranes. Studies have shown that surface-modified nanocellulose exhibits significantly improved dispersion behavior and interfacial compatibility compared to unmodified counterparts, particularly in aqueous and polymeric environments [31,32].

Beyond geometric considerations, nanocellulose displays enhanced chemical reactivity due to the increased accessibility of surface hydroxyl groups and defect sites generated during nanostructuring. These features provide abundant anchoring points for chemical modification, enabling the introduction of charged, hydrophobic, bioactive, or catalytic functionalities. However, the same reactivity can lead to uncontrolled surface interactions if left unmodified, limiting the material’s applicability. Functionalization strategies therefore play a dual role: they not only introduce desired functionalities but also stabilize the nanocellulose surface against environmental and processing-induced degradation. The ability to precisely tailor surface chemistry is particularly critical for applications involving biological interfaces, selective adsorption, or electrochemical processes, where surface interactions govern overall performance [33,34].

At the nanoscale, the relationship between structure and macroscopic properties becomes highly pronounced. Variations in fibril dimensions, crystallinity, surface charge, and network architecture can result in substantial differences in mechanical behavior, permeability, optical transparency, and biological response. Functionalization enables the deliberate tuning of these structure–property relationships by controlling inter-fibrillar interactions and surface-mediated phenomena. For example, functionalized nanocellulose networks can be engineered to exhibit enhanced mechanical robustness, controlled porosity, or stimuli-responsive behavior, which are not attainable with bulk cellulose. This level of control is essential for designing application-specific materials in biomedicine, environmental remediation, and energy systems. Consequently, functionalization should be viewed not as an optional modification step, but as an intrinsic requirement for translating nanocellulose properties into reliable and predictable functional performance [35,36].

## 4. Modification Approaches for Functional Cellulose and Nanocellulose

In this section, the main functionalization pathways are systematically analyzed, including oxidation, esterification, etherification, grafting, and surface/interface modification. These strategies are evaluated within a structure–property–function framework, with particular emphasis on their efficiency, scalability, and environmental trade-offs. Chemical, physical, and surface/interface approaches are compared not only in terms of their mechanisms, but also in how effectively they translate structural changes, such as variations in crystallinity, morphology, and surface chemistry, into measurable functional performance. Particular attention is given to the limitations of each strategy, including energy demand, reagent consumption, process complexity, and their implications for large-scale implementation. These relationships are further interpreted through a unified structure–property–function–process (SPFP) framework, which integrates processing conditions with material design and application requirements.

**Figure 3:**
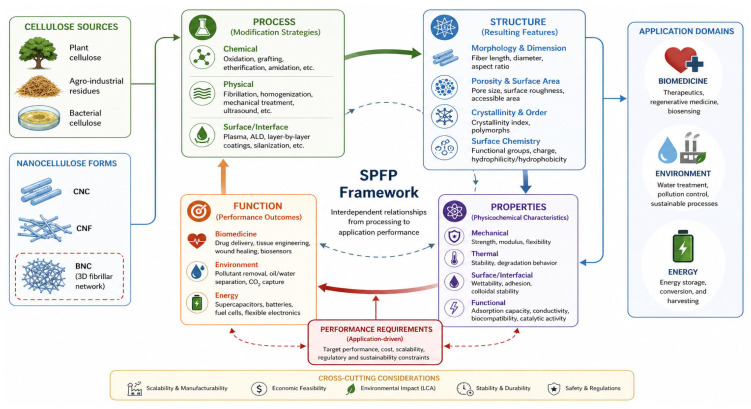
Conceptual representation of the structure–property–function–process (SPFP) framework for functionalized cellulose.

The functional performance of cellulose and nanocellulose is not an intrinsic property of the polymer, but arises from deliberate modification at molecular, surface, and structural levels. While these approaches enable tailored mechanical, interfacial, and functional properties, they often involve competing constraints that limit practical applicability. The interdependent relationships between processing conditions, structural features, physicochemical properties, and functional performance are conceptualized within the SPFP framework, as illustrated in Figure 3.

### 4.1. Chemical Modification of the Cellulose Backbone

Chemical modification of the cellulose backbone represents one of the most effective strategies for enabling controlled functionalization of both bulk cellulose and nanocellulose. Owing to the abundance of hydroxyl groups on the cellulose repeating unit, chemical reactions can be selectively introduced to alter surface charge, solubility, hydrophilicity, and reactivity. This level of molecular control makes chemical approaches particularly attractive for applications requiring precise interfacial tuning. However, such modifications often involve multistep processing, reagent consumption, and potential degradation of structural integrity, which may limit their scalability and sustainability.

**Figure 4:**
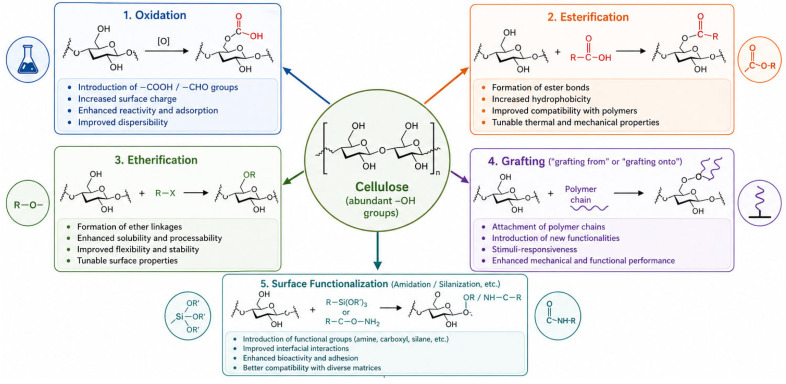
Schematic representation of key chemical functionalization pathways of cellulose, including oxidation, esterification, etherification, grafting, and surface modification.

The main chemical functionalization pathways of cellulose are schematically illustrated in Figure 4, highlighting how different reactions modify hydroxyl groups and lead to distinct functional outcomes.

As shown in Figure 4, chemical modification strategies primarily target hydroxyl groups of cellulose, resulting in changes in surface chemistry, polarity, and reactivity. These transformations enable the tuning of material properties for specific applications, while also introducing trade-offs related to process complexity, chemical consumption, and scalability.

Etherification and esterification are among the most established chemical routes for modifying cellulose. These reactions involve the substitution of hydroxyl groups with ether or ester functionalities, leading to improved solubility, altered intermolecular interactions, and enhanced compatibility with polymer matrices. Industrial derivatives such as hydroxyethyl cellulose and carboxymethyl cellulose illustrate the effectiveness of these approaches. However, while etherification improves processability and dispersion, it may also reduce crystallinity and mechanical strength, particularly at higher degrees of substitution, highlighting a trade-off between functionality and structural stability [6,37].

Oxidation-based modification strategies enable the introduction of reactive functional groups such as aldehydes and carboxylates onto the cellulose surface. These functionalities significantly increase surface charge density and chemical reactivity, enabling improved colloidal stability and enhanced interaction with metal ions or biomolecules. For example, amine-functionalized nanocellulose systems have demonstrated adsorption capacities exceeding 300 mg/g for anionic dyes and heavy metal ions, significantly outperforming unmodified cellulose due to enhanced electrostatic interactions and increased surface reactivity [38,39]. Nevertheless, oxidative treatments may lead to chain scission, reduced degree of polymerization, and increased sensitivity to hydrolytic degradation, which can negatively affect long-term stability in practical applications [40,41].

Grafting strategies involve the covalent attachment of polymer chains or functional molecules onto the cellulose backbone, enabling the creation of hybrid materials with synergistic properties. This approach provides a direct route to introduce stimuli-responsive, antimicrobial, or catalytic functionalities. However, grafting processes are often complex, require controlled reaction conditions, and may suffer from low grafting efficiency or heterogeneous functionalization, limiting reproducibility and large-scale implementation [42]. In addition, amination represents an important modification route, introducing amino groups that enhance adsorption capacity and enable strong interactions with negatively charged species [43].

Overall, chemical modification offers the highest degree of tunability among cellulose functionalization strategies, allowing precise control over surface chemistry and functionality. However, compared to physical and surface modification approaches, it typically involves higher reagent consumption, more complex processing, and the generation of chemical waste. These factors introduce significant trade-offs between performance optimization and sustainability, which remain insufficiently addressed in many studies.

### 4.2. Physical Modification and Structural Engineering of Cellulose and Nanocellulose

Physical modification strategies focus on tailoring the morphology, architecture, and hierarchical organization of cellulose and nanocellulose without altering the covalent structure of the polymer backbone. Unlike chemical modification, physical approaches do not directly alter surface chemistry. Instead, they influence material performance through nanoscale structuring, porosity control, and interfacial organization. These effects are particularly pronounced for nanocellulose, where morphology-driven phenomena strongly govern mechanical behavior, mass transport, and surface accessibility. However, compared to chemical modification, the efficiency of physical approaches is often indirect, as structural changes alone do not necessarily translate into functional performance without additional surface or chemical modification. This limits their standalone applicability and highlights the need for integrated modification strategies.

One of the defining advantages of physical modification lies in its ability to exploit the intrinsic anisotropy and fibrillar nature of cellulose. Processes such as mechanical fibrillation, controlled assembly, and fiber reorganization enable the formation of high-aspect-ratio nanostructures and interconnected networks with large specific surface areas. In nanocellulose aerogel systems, physical structuring through freeze-drying and fibrillar assembly has been reported to increase specific surface area to over 200–400 m^2^/g, thereby significantly improving adsorption efficiency and mass transport properties [44,45]. As a result, physically modified nanocellulose systems often exhibit enhanced mechanical reinforcement, tunable permeability, and improved interaction with surrounding matrices, even in the absence of chemical functionalization [46,47]. While physical modification strategies are often scalable, their energy demand and process control remain important considerations for industrial implementation.

Electrospinning represents a prominent example of morphology-driven physical modification, enabling the transformation of cellulose or regenerated cellulose into continuous nanofibrous mats. By reorganizing cellulose into aligned or randomly oriented nanofibers, electrospinning produces materials with high porosity, interconnected pore networks, and large surface-to-volume ratios. These structural features are particularly advantageous for applications requiring rapid mass transfer, such as filtration membranes, biomedical scaffolds, and energy-related separators. Importantly, electrospinning does not inherently introduce new chemical functionalities. Instead, it creates a structural platform that enhances the efficiency and uniformity of subsequent surface or chemical functionalization [48,49]. Despite these advantages, electrospinning and related structuring techniques require precise control of processing parameters and are often associated with low throughput, which may limit scalability. In addition, their functional contribution remains largely structural, and additional modification steps are typically required to achieve specific chemical or biological activity.

Beyond electrospinning, physical assembly strategies such as freeze-drying, foam forming, and layer-by-layer organization have been widely employed to engineer three-dimensional nanocellulose architectures. Aerogels and foams derived from cellulose nanofibrils exemplify how controlled drying and assembly processes can generate ultra-lightweight materials with high porosity and hierarchical pore structures. These architectures exhibit exceptional specific surface areas and accessibility, which are critical for adsorption-driven applications, catalytic supports, and biointerfaces. The performance of such systems is governed primarily by network topology and fibril interactions rather than by chemical composition alone [50]. Nevertheless, these highly porous structures may suffer from limited mechanical stability and structural collapse under operational conditions, particularly in aqueous or load-bearing environments. This constrains their direct application and necessitates further stabilization strategies.

Physical modification also plays a central role in the formation of dense nanocellulose films, often referred to as nanopapers. Through controlled filtration or casting processes, nanocellulose fibrils self-assemble into compact, layered structures exhibiting high mechanical strength, optical transparency, and barrier properties. These characteristics arise from strong inter-fibrillar hydrogen bonding and nanoscale packing density. While chemically unmodified nanopapers already display remarkable performance, their structural uniformity and surface accessibility make them highly suitable substrates for subsequent functionalization, including surface coating, plasma treatment, or inorganic deposition [51,52]. However, the dense packing that provides mechanical strength may also reduce permeability and limit mass transport, which can be disadvantageous for applications requiring rapid diffusion or high surface accessibility.

Overall, physical modification strategies enable precise control over the structural and morphological features of cellulose-based materials, thereby amplifying the effects of surface chemistry and functionalization. By engineering nanoscale architecture without altering molecular composition, physical approaches provide a versatile and often energy-efficient pathway for optimizing cellulose and nanocellulose performance across biomedical, environmental, and energy-related applications. These strategies should therefore be viewed as complementary to chemical modification, forming an essential component of the integrated functionalization framework discussed in this review. Despite their advantages, physical modification strategies are generally more energy-intensive than chemical approaches, particularly in processes such as mechanical fibrillation and electrospinning. While they avoid the use of chemical reagents, this advantage is often offset by high energy demand and process variability, making their overall sustainability context-dependent rather than inherently superior. This highlights a fundamental trade-off between environmental cleanliness (low chemical input) and energy consumption.

### 4.3. Surface and Interface Modification of Cellulose and Nanocellulose

Surface and interface modification strategies play a pivotal role in tailoring the functional performance of cellulose and nanocellulose without altering the integrity of the polymer backbone. Unlike bulk chemical modification, surface-focused approaches selectively target the outermost molecular layers of cellulose-based materials, enabling precise control over interfacial interactions, wettability, adhesion, and compatibility with surrounding environments. This distinction is particularly important for nanocellulose, where surface phenomena dominate material behavior due to the high surface-to-volume ratio. However, the effectiveness of surface modification is strongly dependent on the accessibility and uniformity of the substrate, which are often governed by prior physical or chemical processing. As a result, surface modification rarely functions as a standalone strategy and typically requires integration with other modification approaches.

One of the major advantages of surface and interface modification lies in its ability to decouple structural integrity from functional performance. By confining chemical or physical changes to the surface, these approaches preserve the inherent mechanical strength and hierarchical architecture of cellulose while enabling the introduction of application-specific functionalities. As a result, surface modification strategies are widely employed to enhance interfacial bonding in composites, regulate mass transport in membranes, and tailor biological interactions in biomedical systems [53,54].

Plasma-based treatments represent a prominent class of surface modification techniques for cellulose and nanocellulose. Exposure to non-thermal plasma generates reactive species capable of selectively introducing oxygen- or nitrogen-containing functional groups at the material surface. These modifications increase surface energy, improve wettability, and enhance adhesion without penetrating into the bulk structure. Plasma-treated nanocellulose surfaces demonstrate improved compatibility with polymer matrices, enhanced dispersion stability, and increased bioactivity. Similarly, conductive surface modification using metallic nanowires or conductive coatings has enabled electrical conductivity values in the range of 10^2^–10^4^ S/m, whereas native cellulose itself is electrically insulating [28,55]. Therefore, plasma processing is considered a versatile and solvent-free approach for interface engineering [56,57]. Despite these advantages, plasma treatment is often limited by shallow penetration depth and potential non-uniformity on complex or dense structures. In addition, maintaining consistent plasma conditions can be challenging, which may affect reproducibility and scalability.

Atomic layer deposition (ALD) offers a complementary strategy by enabling the conformal deposition of ultrathin inorganic coatings onto cellulose and nanocellulose substrates. Owing to its self-limiting surface reactions, ALD provides angstrom-level control over coating thickness and uniformity, even on highly porous and high-aspect-ratio structures. The integration of inorganic layers such as metal oxides onto nanocellulose surfaces has been shown to improve moisture resistance, thermal stability, and mechanical durability, while also enabling new functionalities related to barrier performance, photocatalysis, and electronic interfaces [58,59]. Importantly, ALD modifies interfacial properties without compromising flexibility or nanoscale architecture. However, ALD processes are typically time-consuming and require specialized equipment and controlled environments, which significantly increases production costs. This limits their applicability in large-scale or cost-sensitive applications despite their high precision.

In addition to plasma treatment and ALD, interface modification through coating and immobilization of functional species has been widely explored. The adsorption or covalent attachment of nanoparticles, polymers, or bioactive molecules onto cellulose surfaces enables the creation of hybrid materials with synergistic properties. Such surface-engineered systems combine the sustainability and mechanical robustness of cellulose with the functional attributes of inorganic or polymeric components. This approach has proven particularly effective for developing antimicrobial materials, selective sorbents, and catalytically active supports, where surface accessibility and interfacial chemistry govern performance [60,61]. Nevertheless, the stability of such hybrid systems depends on the strength of interfacial interactions, and leaching or degradation of the functional component may occur under operational conditions. This raises concerns regarding long-term durability and environmental safety.

Overall, surface and interface modification strategies provide a highly effective route for translating the intrinsic properties of cellulose and nanocellulose into application-specific functions. By selectively tailoring surface chemistry and interfacial interactions, these approaches enable fine control over material behavior in complex environments while preserving structural integrity. As such, surface modification represents a critical link between modification strategies and functional performance, bridging the gap between material design and practical application. Although surface modification techniques offer precise control and minimal impact on bulk properties, they are often associated with higher equipment costs and limited scalability. Compared to chemical modification, these approaches may reduce reagent use, but their overall sustainability depends on energy demand, process complexity, and equipment requirements. Consequently, their practical implementation is often constrained by cost, scalability, and reproducibility considerations.

### 4.4. Hybrid and Integrated Modification Strategies

In practical nanocellulose engineering, functional performance is often achieved not through isolated modification routes alone, but through integrated strategies combining chemical, physical, and surface/interface engineering approaches. Such hybrid systems are particularly important for nanocellulose materials, where morphology, surface chemistry, and interfacial organization are strongly interdependent.

Chemo-mechanical approaches represent one of the most widely employed integrated modification strategies. In these systems, chemical pretreatment, such as TEMPO-mediated oxidation or carboxylation, is combined with subsequent mechanical fibrillation to facilitate nanofibril separation, reduce energy consumption, and improve surface accessibility [19,27]. Compared with purely mechanical processing, these hybrid approaches enable the production of more uniform nanostructures with enhanced functionalization efficiency and improved colloidal stability.

Similarly, electrospinning combined with subsequent surface functionalization has been extensively investigated for the fabrication of multifunctional cellulose-based membranes and biomedical scaffolds [62,63]. In such systems, physical structuring creates highly porous fibrillar architectures with large surface areas, while chemical or surface modification introduces targeted functionalities including antibacterial activity, conductivity, hydrophobicity, or selective adsorption behavior. Importantly, hybrid modification strategies often generate synergistic effects that cannot be achieved through isolated chemical or physical modification alone.

Integrated hybrid systems are also increasingly important in energy-related applications, where conductive nanofillers, inorganic nanoparticles, or polymeric coatings are incorporated into nanocellulose networks through combined chemical and physical processing routes [44,64]. These multifunctional systems enable simultaneous optimization of mechanical integrity, interfacial compatibility, conductivity, and electrochemical performance.

Despite their advantages, hybrid modification strategies are often associated with increased process complexity, multistep fabrication, and challenges related to reproducibility, scalability, and environmental sustainability. Therefore, future development of integrated functionalization approaches should focus not only on maximizing material performance, but also on simplifying processing routes, reducing energy consumption, and improving industrial feasibility.

### 4.5. From Modification to Functionalization: Structure–Property–Function Relationships

In line with the terminology adopted in this review, modification strategies define how cellulose is structurally or chemically altered, whereas functionalization refers to the deliberate translation of these modifications into targeted material functions. This distinction highlights that not all modified cellulose materials exhibit comparable functional performance, and underscores the need to explicitly evaluate structure–property–function relationships in material design.

At the nanoscale, even subtle changes in morphology, surface chemistry, or interfacial organization can lead to pronounced differences in macroscopic performance. Physical modification strategies such as fibrillation, electrospinning, or controlled assembly primarily affect parameters including surface area, porosity, and network connectivity. These structural features govern mass transport, mechanical reinforcement, and accessibility of reactive sites, thereby defining the functional potential of the material rather than its immediate functionality. For instance, nanocellulose aerogels with comparable chemical composition may exhibit drastically different adsorption or mechanical behavior depending on pore architecture and fibril alignment [65].

Chemical and surface modification strategies, in contrast, directly influence interfacial interactions and responsiveness by introducing functional groups, charges, or hybrid interfaces. Etherification, oxidation, grafting, and surface coating enable the tuning of hydrophilicity, surface charge density, and affinity toward specific molecules or phases. However, the effectiveness of such chemical modifications is strongly dependent on the underlying structure established during physical modification. High surface accessibility and uniform fibril dispersion are prerequisites for achieving homogeneous functionalization and predictable performance [25,32].

The structure–property–function relationships critically govern the performance of nanocellulose-based systems in biomedicine, environmental remediation, and energy applications. In biomedical scaffolds, for example, cellular response is governed not only by surface chemistry but also by nanoscale topography, porosity, and mechanical compliance. Similarly, in sorption-based environmental applications, adsorption capacity and selectivity depend on the interplay between surface functional groups and accessible surface area. In energy-related applications, including separators and electrode components, ionic transport and mechanical stability depend on both network architecture and interfacial chemistry. These properties cannot be explained by chemical composition alone [66,67,68].

This interdependence indicates that functionalization is a multi-level design process integrating physical structuring, chemical modification, and surface engineering. Effective functional materials emerge when physical structuring, chemical modification, and surface engineering are combined in a rational manner. This hierarchical approach enables the decoupling of competing requirements, such as mechanical robustness and chemical reactivity, and allows cellulose-based materials to be tailored for specific operational environments.

Nanocellulose acts as an enabling platform in which structural features amplify the effects of chemical and surface modification, allowing relatively small interfacial changes to produce significant functional outcomes. Consequently, understanding and controlling structure–property–function relationships is central to advancing cellulose and nanocellulose from laboratory-scale materials toward reliable components in real-world biomedical, ecological, and energy systems. A critical comparison of modification strategies, including their structural impact, performance benefits, and practical limitations, is summarized in Table 3.

Table 3 demonstrates that no modification strategy is universally optimal. Chemical approaches offer high tunability but involve significant reagent consumption and potential structural degradation. Physical methods enhance morphology and accessibility without chemical input, yet are often energy-intensive. Surface/interface techniques provide precise interfacial control but are constrained by cost and scalability.

These comparisons reveal several unresolved gaps. First, modification strategies are rarely evaluated under comparable conditions, limiting cross-study benchmarking. Second, structure–property relationships are often described qualitatively rather than quantitatively. Third, scalability, cost, and environmental impact remain insufficiently integrated into performance evaluation.

To address these limitations, it is necessary to move beyond the conventional classification of modification strategies into chemical, physical, and surface approaches and adopt a unified structure–property–function–process (SPFP) framework. Within this perspective, functionalization is not treated as an isolated step, but as part of an interconnected system in which processing conditions determine structural features, which in turn govern physicochemical properties and ultimately define functional performance. Importantly, this relationship is bidirectional: performance requirements also constrain the selection of modification strategies and processing routes.

This framework provides a basis for the rational design of functionalized cellulose systems by explicitly integrating material structure, targeted properties, processing complexity, scalability, and environmental impact. Rather than considering modification strategies independently, the SPFP approach enables their systematic comparison and combination based on application-specific requirements and inherent trade-offs.

Despite the widespread perception of cellulose as an inherently sustainable material, its environmental performance cannot be assumed a priori and must be evaluated across its full life cycle. Chemical functionalization routes frequently rely on reactive reagents, organic solvents, and multistep processing, which may generate hazardous waste and increase environmental burden. In addition, nanocellulose production, particularly via mechanical fibrillation or chemo-mechanical treatments, can be highly energy-intensive, partially offsetting the benefits of renewable feedstocks. End-of-life considerations remain insufficiently addressed, as hybrid systems incorporating synthetic polymers, cross-linking agents, or inorganic components may exhibit reduced biodegradability and limited recyclability. Therefore, sustainability must be critically assessed across processing, application, and disposal stages.

Therefore, the following section critically examines how modification-induced structural features translate into functional performance across biomedical, environmental, and energy systems, with emphasis on benchmarking, trade-offs, and practical implementation. The SPFP framework provides not only a conceptual basis for comparing modification strategies, but also a practical tool for understanding how processing-induced structural features determine functional performance in specific application domains. In this context, biomedical, environmental, and energy-related systems can be interpreted as distinct manifestations of structure–property–function relationships, where the effectiveness of functionalized cellulose depends on balancing interfacial activity, mechanical stability, transport properties, and scalability constraints. Therefore, the following section applies the SPFP perspective to critically analyze how modification-induced structural and physicochemical characteristics translate into measurable application performance and practical limitations.

## 5. Applications of Functionalized Cellulose and Nanocellulose

The functional performance of cellulose and nanocellulose-based materials in practical applications is governed by the interplay between structure, physicochemical properties, and resulting functional behavior. As established in Section 3, modification strategies alter cellulose at multiple levels, molecular, surface, and structural, thereby defining parameters such as surface area, charge density, porosity, mechanical integrity, and interfacial reactivity.

In this context, the structure–property–function relationship serves as a critical analytical framework for evaluating material performance. Rather than treating applications as isolated outcomes, this section examines how specific structural features induced by functionalization translate into measurable performance in biomedical, environmental, and energy systems. Particular attention is given to cases where expected performance enhancements are limited by competing factors such as stability, scalability, and cost.

As discussed in previous sections and summarized in Table 4 and Table 5, the functional outcomes achieved through tailored modification strategies directly determine the suitability of functionalized cellulose for biomedical, environmental, and energy-related applications. Functionalized cellulose has been increasingly explored across multiple application domains due to its biodegradability, high mechanical strength, chemical stability and modification capabilities. Depending on the modification strategy (chemical or physical), cellulose acquires new properties, such as improved adsorption capacity, biocompatibility, conductivity, and catalytic activity, which makes it a promising material for biotechnology [8], ecology [9], medicine [69], energy [70]. To provide a structured overview of how functionalization strategies translate into performance across different application domains, representative examples and key challenges are summarized in Table 4.

The comparison presented in Table 4 highlights that the performance of functionalized cellulose is strongly application-dependent and governed by trade-offs between functional efficiency, stability, and scalability. While modification strategies enable targeted performance improvements, they often introduce limitations related to environmental sensitivity, regeneration, or intrinsic material properties. These results emphasize that functionalized cellulose should not be viewed as a universally superior material platform, but rather as a system whose effectiveness depends on careful optimization of structure–property–function relationships.

Despite its versatility, functionalized cellulose remains constrained by several critical limitations. In energy-related applications, low intrinsic electrical conductivity often necessitates the incorporation of conductive additives, increasing complexity and cost. In aqueous and biological environments, hydrothermal and structural stability can be insufficient, particularly for highly porous or extensively modified systems. In addition, many functionalization strategies involve multistep processing, high energy input, or the use of reactive chemicals, which complicate scalability and may reduce overall sustainability. These factors must be carefully considered when assessing the real-world applicability of cellulose-based materials.

### 5.1. Application in Biomedicine

The performance of cellulose-based materials in biomedical applications is primarily determined by their structural features, such as porosity, surface chemistry, and mechanical compliance, which directly influence cell-material interactions and drug release behavior.

As summarized in Table 4, biomedical applications primarily rely on hydrogel formation and surface grafting to achieve biocompatibility and controlled bioactivity. Functionalized cellulose is a promising class of biomaterials for medicine due to its biocompatibility, biodegradability, high mechanical strength and ability to be modified. In recent years, its use has expanded significantly, covering such areas as the development of hydrogels, drug delivery systems, tissue engineering, biosensors and antibacterial coatings [71,72,73]. These applications directly reflect the structure–property–function relationships discussed in Section 3, where surface chemistry, porosity, and mechanical compliance govern biological response. Compared to conventional biomaterials such as collagen, alginate, and synthetic polymers, cellulose-based systems generally exhibit comparable biocompatibility and mechanical stability. However, their intrinsic bioactivity and ability to actively regulate cell behavior are often lower, making their performance more dependent on surface modification and scaffold architecture.

One of the key areas of application of functionalized cellulose is the creation of hydrogels, which are used as wound-healing dressings, drug carriers and biopolymer bases for contact lenses [74]. Due to their high hydrophilicity and ability to retain a significant amount of water, such hydrogels promote rapid wound healing, preventing infection and providing controlled release of active substances. In addition, the introduction of antibacterial components such as silver (Ag) [75], zinc oxide (ZnO) [76] and chitosan [77] into their composition can improve their effectiveness in the treatment of infected wounds and burns.

A representative example involves Ca^2+^-cross-linked nanofibrillated cellulose (NFC) hydrogels prepared from TEMPO-oxidized cellulose [78]. These systems demonstrate enhanced cell adhesion, proliferation, and migration of fibroblasts and keratinocytes, and have shown improved wound healing performance in in vivo models, including accelerated epithelialization and tissue regeneration. Such results highlight the potential of nanocellulose-based hydrogels for biomedical applications, particularly in wound healing. However, their performance remains strongly dependent on cross-linking conditions, and long-term stability under physiological environments is still insufficiently evaluated.

Recent studies (2024–2025) demonstrate that functionalized cellulose is increasingly explored as a platform for drug delivery systems [79,80]. These properties enable the design of carriers capable of controlled drug release and reduced systemic toxicity. For example, functionalized cellulose nanocrystals have been used to develop colon-targeted delivery systems, where drug release is triggered by enzymatic conditions, allowing minimal release in the upper gastrointestinal tract and enhanced release in the colon [81]. However, cellulose-based systems differ from established drug delivery platforms such as liposomes and polymeric nanoparticles. They mainly rely on structural control rather than active targeting mechanisms. As a result, their performance remains strongly dependent on surface functionalization and environmental conditions, which may limit precise control over drug release and targeting efficiency.

In the field of tissue engineering, functionalized cellulose is used to create biocompatible scaffolds on which cells can develop [82,83,84]. These structures play an important role in the regeneration of bone, cartilage, skin and nerve tissue. The development of 3D printing technology has made it possible to use modified cellulose as ink for bioprinting, which opens up new prospects in the creation of personalized implants and biomaterials. The research describes a method for developing biofunctionalized cellulose acetate (CA) nanoscaffolds aimed at improving heart valve tissue engineering [85]. Functionalized cellulose scaffolds have also been explored for tissue engineering, where surface modification enhances cell adhesion and proliferation. Such systems aim to mimic extracellular matrix behavior and improve integration with biological tissues. However, their performance remains dependent on surface functionalization efficiency and long-term biological stability.

Despite these advances, cellulose-based biomaterials still face limitations related to long-term biocompatibility, immunogenic response, and the potential cytotoxicity of incorporated components. In addition, their performance remains strongly dependent on modification strategies, which complicates direct comparison with established biomaterials and highlights the need for further validation.

### 5.2. Environmental Applications

Recent studies (2024–2025) highlight the growing interest in cellulose-based sorbents for environmental remediation. In environmental applications, adsorption performance is largely governed by surface functional groups, pore structure, and accessibility, which determine interaction mechanisms with pollutants.

Environmental applications focus on amination and oxidation strategies to maximize adsorption capacity, although regeneration and selectivity remain critical challenges (Table 4). Functionalized cellulose plays an important role in environmental technologies, offering sustainable solutions for water and air purification, pollutant sorption, development of biodegradable packaging materials and reduction of man-made load on the environment [86,87]. Due to biodegradability, high sorption capacity and modification possibilities, cellulose is used in the creation of environmentally friendly sorbents, filtration membranes and sustainable polymer composites.

A representative approach involves amine-functionalized cellulose, where surface modification increases adsorption efficiency toward anionic dyes [43]. The introduction of amino groups enhances electrostatic interactions and improves selectivity. However, adsorption performance remains sensitive to ionic strength and water composition, which may limit practical applicability.

Cellulose-based aerogels have demonstrated high oil absorption capacity and selectivity for oil-water separation [88]. However, solvent-intensive processing and freeze-drying limit scalability and industrial feasibility.

The study investigates the stability of amine-functionalized cellulose (AEAPDMS-NFC) as a sorbent for direct air capture of CO_2_ during temperature-vacuum-swing (TVS) cycling. Amine-functionalized cellulose has been investigated for CO_2_ capture, showing promising cyclic stability [89]. The method focused on understanding the mechanical and chemical stability of the sorbent over repeated operational conditions. Although long-term cyclic stability was demonstrated, adsorption capacity under ambient CO_2_ concentrations remains a key bottleneck for large-scale deployment. However, adsorption capacity under ambient conditions remains a key limitation for large-scale applications.

A representative approach involves the functionalization of nanocrystalline cellulose with amine groups to enhance arsenic removal from aqueous systems [90]. This modification introduces positively charged sites that promote electrostatic interaction with negatively charged arsenic species, resulting in high adsorption efficiency. While such systems demonstrate strong removal performance under controlled conditions, their effectiveness remains dependent on water composition and operational parameters, which may limit performance in complex real-world environments.

Compared with commercial activated carbon, cellulose-based adsorbents are more attractive in terms of renewability, biodegradability, and surface functionalization potential. However, activated carbon remains superior in industrial maturity, broad pollutant applicability, regeneration infrastructure, and operational stability. Therefore, functionalized cellulose should not be presented as a direct universal replacement for activated carbon, but rather as a promising alternative for targeted applications where selectivity, biodegradability, or low-cost biomass sourcing are prioritized.

Overall, the reviewed studies demonstrate significant progress in the application of functionalized cellulose for environmental remediation. In particular, cellulose-based systems show strong potential for the removal of toxic pollutants from water sources. Moreover, the sorbents demonstrated favorable reusability, which is of great importance for practical applications in large-scale remediation efforts. Overall, functionalized cellulose demonstrates strong potential for environmental remediation, particularly when surface chemistry is tailored toward specific pollutants. However, most reported results are obtained under controlled laboratory conditions, whereas real wastewater contains competing ions, organic matter, variable pH, and fouling agents. These factors can reduce adsorption efficiency and complicate regeneration. Thus, the key challenge is not only achieving high adsorption capacity, but also proving long-term stability, regeneration efficiency, and competitiveness with established sorbents under realistic operating conditions.

An additional challenge in environmental applications is the regeneration and long-term stability of functionalized cellulose sorbents. Although many cellulose-based adsorbents demonstrate high initial adsorption efficiency, repeated adsorption–desorption cycles may lead to structural degradation, loss of active functional groups, pore collapse, and reduced sorption capacity. Regeneration efficiency strongly depends on the type of functionalization, desorption conditions, and operational environment. In complex wastewater systems, fouling by organic matter and competing ions may further reduce recyclability and adsorption selectivity. Therefore, improving regeneration stability and maintaining adsorption performance over multiple cycles remain essential requirements for large-scale environmental implementation of functionalized cellulose materials.

### 5.3. Energy-Related Applications

In energy-related systems, hybrid interfaces dominate functionalization strategies, but trade-offs between conductivity, cycling stability, and energy density persist (Table 4). Functionalized cellulose plays a key role in the development of energy-efficient and environmentally friendly technologies, offering sustainable solutions for energy accumulation, conversion and storage [91,92,93]. Due to its high mechanical strength, porous structure, biodegradability and chemical modification capabilities, cellulose is widely used in batteries, supercapacitors, fuel cells and photovoltaic devices.

The fabrication of Ag nanowire (Ag NW)-functionalized cellulose textiles is typically achieved through a simple dipping and drying process, resulting in conductive coatings with low sheet resistance, high mechanical stability, and good chemical durability [94]. These materials are attractive for applications in wearable electronics and energy storage devices due to their flexibility and conductivity. However, such systems rely on the incorporation of highly conductive additives, which increases material cost and may compromise sustainability, limiting their scalability.

Hybrid proton exchange membranes (PEMs) based on cellulose whiskers are commonly prepared via surface functionalization followed by incorporation into polymer matrices such as sulfonated polysulfone [95]. These systems exhibit improved proton conductivity and membrane performance, depending on the type of functional groups introduced. However, despite these improvements, such hybrid systems remain limited by long-term chemical stability and performance degradation under realistic operating conditions.

The paper outlines several methodologies for fabricating multifunctional cellulose paper suitable for optoelectronic applications [96]. Key techniques include inkjet and screen printing, which allow for the direct application of nanoparticle colloidal solutions onto paper surfaces, enabling the creation of SERS (Surface Enhanced Raman Scattering) substrates. These techniques are praised for their affordability and the ability to produce customized shapes for SERS-active regions. Additionally, methods such as dip coating and spin coating are discussed for creating thin films, with dip coating providing uniform layers but at the cost of material waste. The authors also mention innovative approaches like the “pen on paper” method, which utilizes a fountain pen filled with metal nanoparticle ink to directly create plasmonic areas on paper without requiring special equipment or extensive training. Overall, these methods emphasize the adaptability of cellulose as a substrate for low-cost, flexible electronic devices while exploring various fabrication processes that enhance their performance in light harvesting and sensing applications.

When compared with established energy technologies such as lithium-ion batteries and Nafion-based proton exchange membranes, cellulose-based materials generally exhibit lower energy density, electrical conductivity, and long-term operational stability. Despite the growing interest in functionalized cellulose for energy-related applications, several critical limitations must be acknowledged when benchmarking these materials against conventional energy technologies. In electrochemical energy storage systems, cellulose-based electrodes and supercapacitors typically exhibit lower energy density than carbon-based materials or metal oxides. This limitation is mainly associated with low electrical conductivity and restricted charge-storage mechanisms. While functionalization and hybridization with conductive fillers such as silver nanowires or carbon nanostructures can significantly enhance conductivity, this often introduces trade-offs related to material cost, interfacial stability, and recyclability. In proton exchange membranes and fuel cell components, cellulose-based systems demonstrate promising proton conductivity and mechanical flexibility; however, long-term durability, chemical stability under acidic or oxidative conditions, and performance retention over extended operating cycles remain key challenges. Furthermore, many fabrication techniques employed for energy-oriented cellulose materials, including nanowire deposition, surface modification, and printing-based approaches, face scalability and process reproducibility issues that limit industrial implementation. Compared indirectly with established inorganic or polymer-based energy materials, functionalized cellulose offers clear advantages in sustainability, flexibility, and lightweight design, but further optimization of conductivity, cycle life, and manufacturing efficiency is required before these systems can compete in high-performance energy storage and conversion devices.

To further integrate the structure–property–function relationships across different application domains, a cross-domain mapping is presented in Table 5.

Table 5 highlights how specific structural features govern property development and determine functional performance in biomedical, environmental, and energy applications. This synthesis demonstrates that similar structural characteristics may lead to different outcomes depending on the application context, while also revealing common limitations related to stability, scalability, and environmental conditions.

To further strengthen cross-study comparison and provide quantitative benchmarking of functionalized cellulose systems, representative performance indicators reported for different modification strategies and application domains are summarized in Table 6. The presented data illustrate how structural modification and functionalization directly influence adsorption behavior, conductivity, biocompatibility, and operational stability, while also highlighting key practical limitations.

The comparison presented in Table 6 demonstrates that the effectiveness of functionalized cellulose strongly depends on the targeted application and modification strategy. Biomedical systems generally exhibit high biocompatibility and favorable biological response, whereas environmental applications are characterized by high adsorption performance but limited regeneration stability under complex operating conditions. In energy-related systems, conductivity enhancement often requires incorporation of conductive fillers, which improves electrochemical performance but increases material complexity and cost. These comparisons further confirm that no single functionalization strategy universally satisfies all performance requirements, emphasizing the importance of balancing functionality, stability, scalability, and sustainability within the proposed SPFP framework.

Overall, the reviewed energy-related applications demonstrate that functionalized cellulose can successfully operate as a structural, interfacial, or active component in emerging energy technologies. However, the performance of these systems is strongly constrained by trade-offs between sustainability, conductivity, durability, and manufacturing complexity. While laboratory-scale studies highlight the versatility of cellulose-based platforms, their translation into competitive energy devices requires a deeper understanding of structure-function optimization, long-term stability, scalability, and economic feasibility. These aspects remain critical bottlenecks and motivate a broader discussion on innovation potential, challenges, and scaling issues, which is addressed in the following section. In energy-related applications, regeneration and long-term operational durability also remain important challenges. Repeated charge–discharge cycles, thermal fluctuations, and interfacial degradation may gradually reduce conductivity, structural integrity, and electrochemical performance of cellulose-based systems. In hybrid materials, instability at the interface between cellulose and conductive fillers can further accelerate performance loss during prolonged operation. Consequently, future studies should focus not only on improving initial electrochemical characteristics, but also on enhancing cycling stability, recyclability, and long-term reliability under realistic operational conditions.

## 6. Innovation Potential, Challenges, and Future Research Directions

Functionalized cellulose has emerged as a versatile and sustainable platform for the development of advanced materials that integrate responsiveness, mechanical robustness, and environmental compatibility. Recent research trends indicate a clear shift from single-function cellulose derivatives toward multifunctional systems capable of sensing, actuation, energy storage, and controlled interaction with biological and environmental media. These advances are largely enabled by the precise control of surface chemistry, hierarchical structure, and hybrid interfaces discussed in the preceding sections.

One of the most dynamic innovation directions involves stimuli-responsive and smart cellulose-based materials. Cellulose functionalization enables responsiveness to pH, temperature, light, and humidity, which is essential for applications in sensing, drug delivery, and adaptive packaging. Comprehensive analyses of cellulose-based fluorescent and smart materials demonstrate how functional groups and hybrid nanostructures enable optical and electrical signal transduction in response to environmental changes [97]. Similarly, cellulose-based flexible functional materials have been shown to support intelligent electronic systems, combining mechanical flexibility with sensing and signal-processing capabilities [98]. Smart paper platforms integrating cellulose microfibers with carbon nanotubes further illustrate how cellulose can serve as a mechanically robust yet electronically active substrate for multifunctional sensing applications [99]. Despite these advances, challenges remain in achieving long-term signal stability, reversible responsiveness, and reliable performance under cyclic loading and real-use conditions.

Another important research direction is the development of cellulose-based materials for energy storage and conversion. Recent reviews highlight the growing role of cellulose-derived smart materials in supercapacitors, batteries, and energy conversion devices, where nanocellulose networks act as flexible scaffolds for conductive and electroactive phases [100]. The primary limitation in this area remains the relatively low energy density and limited cycling stability of cellulose-based systems compared to conventional carbon- or metal-based electrodes. Addressing this gap requires optimization of hybrid architectures, improved interfacial bonding between cellulose and conductive fillers, and rational design of hierarchical pore structures to facilitate charge transport.

Cellulose functionalization also enables pH-, temperature-, and light-responsive systems with direct relevance to environmental and biomedical technologies. pH-sensitive cellulose-based hydrogels have demonstrated high efficiency in pollutant removal by combining responsive swelling behavior with tunable surface charge, allowing selective interaction with contaminants under varying conditions [101]. Temperature-responsive cellulose thin films further expand the functional scope of cellulose by enabling reversible changes in surface properties, which are relevant for smart coatings and packaging applications [102]. Light-responsive cellulose nanocrystal composites exhibit mechanically robust behavior combined with ultraviolet-triggered responses, illustrating how external stimuli can be leveraged to activate functional performance on demand [103]. However, the scalability and durability of such responsive systems remain key challenges, particularly under prolonged exposure to fluctuating environmental conditions.

The integration of cellulose with carbon-based nanomaterials, such as graphene and carbon nanotubes, represents another major innovation pathway. Recent studies and reviews demonstrate that cellulose–graphene hybrid aerogels and nanocomposites exhibit enhanced mechanical strength, electrical conductivity, and multifunctionality, making them suitable for applications ranging from water treatment to flexible electronics [104,105]. While these hybrids significantly expand the functional envelope of cellulose, they also introduce trade-offs related to cost, processing complexity, and recyclability. Maintaining the environmental advantages of cellulose while incorporating high-performance nanofillers remains a critical design challenge.

In the biomedical domain, cellulose-based biosensors and diagnostic platforms have attracted sustained attention. Advances in cellulose-based electrochemical and optical biosensors demonstrate high sensitivity toward clinically relevant biomarkers, enabling applications in point-of-care diagnostics and early disease detection [106,107]. Recent reviews on nanotechnology-enabled diagnostics highlight the importance of cellulose substrates in developing affordable, portable, and biodegradable testing platforms for infectious diseases, particularly in resource-limited settings [108,109]. Nevertheless, issues related to batch-to-batch reproducibility, interference from complex biological matrices, and long-term storage stability continue to limit large-scale deployment.

Functionalized cellulose has also gained prominence as a sustainable alternative for food packaging and disposable materials. Recent progress in the functionalization of cellulose nanofibers for active food packaging demonstrates improvements in barrier properties, antimicrobial activity, and mechanical performance [110,111]. Broader reviews of cellulose derivatives in packaging applications confirm their potential to replace petroleum-based polymers while reducing environmental impact [112]. However, industrial implementation remains constrained by production costs, compatibility with existing processing lines, and the need for comprehensive life-cycle assessments to validate environmental benefits at scale. Importantly, these findings demonstrate that the sustainability of cellulose-based materials cannot be assumed a priori, but must be validated through comprehensive life-cycle assessment.

Finally, flexible and wearable electronics represent one of the most forward-looking application domains for functionalized cellulose. Cellulose-based flexible sensors and substrates enable lightweight, breathable, and biocompatible devices for health and environmental monitoring [113]. Cellulose nanofibers have been successfully employed as substrates for biodegradable moisture sensors, demonstrating reliable performance combined with mechanical flexibility [114]. Emerging cellulose-derived materials further support the development of wearable sensors capable of monitoring physiological and environmental parameters in real time [115]. Despite these advances, achieving long-term durability, stable electrical performance, and integration with existing electronic architectures remains a central research challenge.

Overall, the innovation potential of functionalized cellulose is substantial, but its translation into industrially viable technologies requires addressing several cross-cutting challenges. These include the cost and complexity of multistep functionalization processes, sensitivity of performance to environmental conditions, limited standardization of material characterization, and the need for scalable, energy-efficient manufacturing routes. Future research should prioritize structure-function optimization under realistic operating conditions, hybrid material design with minimal environmental trade-offs, and rigorous benchmarking against established materials. By aligning material innovation with scalability, reproducibility, and sustainability considerations, functionalized cellulose can evolve from a promising research platform into a cornerstone of next-generation sustainable technologies.

## 7. Conclusions

This review highlights that the performance of functionalized cellulose and nanocellulose is not determined by modification strategies alone, but by the complex interplay between structure, properties, and function. While chemical, physical, and surface modification approaches enable targeted tuning of material behavior, their effectiveness is inherently constrained by trade-offs related to stability, scalability, and environmental impact.

Across biomedical, environmental, and energy applications, functionalized cellulose demonstrates clear advantages in terms of renewability, structural versatility, and compatibility with diverse functionalization routes. However, these advantages do not universally translate into superior performance. In many cases, cellulose-based systems remain limited by lower intrinsic conductivity, insufficient long-term stability in aqueous or physiological environments, and strong dependence on additional functionalization to achieve application-specific performance.

A key challenge in the field is the lack of systematic benchmarking against established materials, which complicates the evaluation of real-world competitiveness. Future research should therefore prioritize the development of standardized comparison frameworks, scalable and energy-efficient processing routes, and improved performance under realistic operating conditions. In particular, enhancing durability, regeneration efficiency, and interfacial stability will be critical for practical implementation.

Compared to other biopolymer platforms, cellulose offers a unique combination of hierarchical structure, high surface functionality, and mechanical robustness, enabling multi-scale design from molecular to macroscopic levels. However, the future relevance of cellulose will depend not only on its inherent properties. It will also depend on the ability to overcome current limitations and achieve competitive performance in technologically demanding applications. The proposed structure–property–function–process framework provides a basis for the rational design of functionalized cellulose systems by integrating performance requirements with processing and sustainability constraints.

Overall, functionalized cellulose should be regarded not as a universal replacement for conventional materials, but as a strategically tunable platform whose performance depends on the rational integration of structure, function, and processing constraints. In addition, future studies should incorporate life-cycle assessment and environmental impact analysis to critically validate the sustainability claims of functionalized cellulose systems.

## Figures and Tables

**Table 1:** Comparison of cellulose sources for nanocellulose production.

Cellulose Source	Typical Nanocellulose Type	Key Structural Features	Functional Advantage	Key Limitation	Scalability/Cost	Main Application Domains
Wood pulp/plant fibers	CNCs, CNFs	Hierarchical fibrils, semicrystalline	High OH accessibility, well-established processing	Requires chemical pretreatment, energy-intensive fibrillation	High scalability, moderate cost	Composites, membranes, energy
Agro-industrial residues	CNCs, CNFs	Variable crystallinity, residual lignin	Low cost, sustainable feedstock	Structural heterogeneity, purification required	High scalability, low cost	Sorption, filtration, eco-materials
Bacterial cellulose	BNC	Pure 3D nanofibrillar network	High purity, uniform structure, excellent biocompatibility	High production cost, limited large-scale yield	Low scalability, high cost	Biomedicine, biointerfaces

**Table 2:** Comparative analysis of physicochemical properties and functional implications of CNCs, CNFs, and BNC.

Parameter	CNCs	CNFs	BNC
Morphology	Rigid rod-like particles	Flexible fibrillar network	3D nanofibrillar network
Structural advantage	High crystallinity, well-defined geometry	High aspect ratio, network connectivity	Highly uniform and pure structure
Functional benefit	Good reinforcement, predictable behavior	High surface area, tunable porosity	Excellent biocompatibility, stable interfaces
Key limitation	Brittleness, limited flexibility	Structural heterogeneity, difficult control	High production cost, limited scalability
Application relevance	Composites, reinforcement materials	Filtration, adsorption, membranes	Biomedical scaffolds, biointerfaces

**Table 3:** Critical comparison of modification strategies in cellulose functionalization.

Modification Strategy	Structural Effect	Property Enhancement	Key Advantage	Major Limitation	Scalability	Environmental Impact
Chemical (oxidation, grafting)	Introduces functional groups, alters surface chemistry	Increased reactivity, adsorption, bioactivity	High tunability of surface chemistry	High chemical consumption, possible loss of crystallinity	Moderate–low	Use of reagents, waste generation
Physical (fibrillation, electrospinning)	Changes morphology, increases surface area	Improved mechanical properties, porosity	No chemical modification required	High energy consumption, limited control at molecular level	Moderate	Energy-intensive processes
Surface/interface (plasma, ALD)	Modifies outer layer without bulk disruption	Enhanced wettability, adhesion, stability	Precise surface control	Equipment complexity, high cost	Low–moderate	Generally cleaner, but energy-dependent

**Table 4:** Comparative analysis of functionalized cellulose applications and associated limitations.

Application Field	Functionalization Strategy	Functional Advantage	Key Performance Metrics	Key Limitation	Underlying Constraint
Biomedicine	Hydrogel formation, surface grafting	Biocompatibility, controlled drug release	Cell viability, release kinetics	Long-term stability, variability of biological response	Sensitivity to physiological conditions, degradation control
Environment	Amination, oxidation	High adsorption selectivity, tunable surface charge	Adsorption capacity, selectivity	Regeneration efficiency, fouling, competing ions	Dependence on water composition and surface chemistry
Energy	Hybrid interfaces, conductive coatings	Structural flexibility, lightweight design	Electrical conductivity, cycling stability	Low energy density, limited durability	Intrinsic low conductivity, interface instability

**Table 5:** Cross-domain mapping of structure–property–function relationships in functionalized cellulose systems.

Structural Feature	Property Driver	Biomedical Relevance	Environmental Relevance	Energy Relevance	Limitation
Surface charge (–COOH, –NH_2_)	Electrostatic interactions	Drug loading, cell interaction	Adsorption of ions/dyes	Ion transport	Sensitivity to pH and ionic strength
High porosity/surface area	Mass transfer efficiency	Tissue scaffolds	Filtration, sorption	Electrodes, separators	Structural instability
Hierarchical fibrillar network	Mechanical strength, flexibility	Wound healing matrices	Membrane durability	Flexible devices	Processing complexity
Conductive modification	Electron transport	Biosensors	Limited	Energy storage	Requires additives

**Table 6:** Representative performance indicators of functionalized cellulose systems in different application domains.

Application Field	Functionalization Strategy	Representative Performance Indicator	Typical Value/Range	Main Limitation
Biomedical	TEMPO-oxidized NFC hydrogels	Cell viability	>85–95%	Limited long-term physiological stability
	Ag-modified cellulose hydrogels	Antibacterial efficiency	>90% bacterial inhibition	Potential cytotoxicity of metal nanoparticles
Environmental	Amine-functionalized nanocellulose	Adsorption capacity for dyes	150–450 mg/g	Reduced efficiency in complex wastewater
	Cellulose aerogels	Oil absorption capacity	20–80 g/g	Structural collapse after repeated cycles
	Functionalized cellulose for CO_2_ capture	CO_2_ adsorption capacity	1–3 mmol/g	Limited adsorption under ambient conditions
Energy	Ag nanowire/cellulose composites	Electrical conductivity	10^2^–10^4^ S/m	High cost of conductive fillers
	Cellulose-based proton exchange membranes	Proton conductivity	10^−3^–10^−2^ S/cm	Long-term chemical instability
	Cellulose supercapacitor electrodes	Cycling stability	>80% retention after 1000 cycles	Lower energy density than conventional materials

## Data Availability

No new data were created or analyzed in this study.

## References

[1] Marinho E. (2025). Cellulose: A Comprehensive Review of Its Properties and Applications. Sustain. Chem. Environ..

[2] Antony Jose S., Cowan N., Davidson M., Godina G., Smith I., Xin J., Menezes P.L. (2025). A Comprehensive Review on Cellulose Nanofibers, Nanomaterials, and Composites: Manufacturing, Properties, and Applications. Nanomaterials.

[3] Sonowal D., Wani K.M. (2025). Comprehensive Review of Cellulose Nanocrystals: Preparation, Properties, Modifications and Applications. Bull. Natl. Res. Cent..

[4] Jedvert K., Heinze T. (2017). Cellulose Modification and Shaping—A Review. J. Polym. Eng..

[5] Aziz T., Farid A., Haq F., Kiran M., Ullah A., Zhang K., Li C., Ghazanfar S., Sun H., Ullah R. (2022). A Review on the Modification of Cellulose and Its Applications. Polymers.

[6] Roy D., Semsarilar M., Guthrie J.T., Perrier S. (2009). Cellulose Modification by Polymer Grafting: A Review. Chem. Soc. Rev..

[7] Bangar S.P., Harussani M.M., Ilyas R.A., Ashogbon A.O., Singh A., Trif M., Jafari S.M. (2022). Surface Modifications of Cellulose Nanocrystals: Processes, Properties, and Applications. Food Hydrocoll..

[8] Beims R.F., Arredondo R., Sosa Carrero D.J., Yuan Z., Li H., Shui H., Zhang Y., Leitch M., Xu C.C. (2022). Functionalized Wood as Bio-Based Advanced Materials: Properties, Applications, and Challenges. Renew. Sustain. Energy Rev..

[9] Kasbaji M., Mennani M., Oubenali M., Ait Benhamou A., Boussetta A., Ablouh E.-H., Mbarki M., Grimi N., El Achaby M., Moubarik A. (2023). Bio-Based Functionalized Adsorptive Polymers for Sustainable Water Decontamination: A Systematic Review of Challenges and Real-World Implementation. Environ. Pollut..

[10] Darmenbayeva A., Rajasekharan R., Massalimova B., Bektenov N., Taubayeva R., Bazarbaeva K., Kurmanaliev M., Mukazhanova Z., Nurlybayeva A., Bulekbayeva K. (2024). Cellulose-Based Sorbents: A Comprehensive Review of Current Advances in Water Remediation and Future Prospects. Molecules.

[11] Al-Zu’bi M., Fan M. (2025). Nanocellulose Technologies: Production, Functionalization, and Applications in Medicine and Pharmaceuticals—A Review. J. Biomed. Mater. Res..

[12] Rafee S.M.I.A., Alim M.S.-U., Alam S., Salem K.S. (2025). Nanocellulose Polymorphs for Biomedical Applications: Recent Advances, Prospects and Challenges—A Review. Carbohydr. Polym. Technol. Appl..

[13] Thomas S.P. (2025). A Brief Review on Extraction and Characterization of Nanocellulose from Date Palm Biomass. J. King Saud. Univ. Eng. Sci..

[14] Klemm D., Heublein B., Fink H., Bohn A. (2005). Cellulose: Fascinating Biopolymer and Sustainable Raw Material. Angew. Chem. Int. Ed..

[15] Moon R.J., Martini A., Nairn J., Simonsen J., Youngblood J. (2011). Cellulose Nanomaterials Review: Structure, Properties and Nanocomposites. Chem. Soc. Rev..

[16] Thomas B., Raj M.C., B A.K., H R.M., Joy J., Moores A., Drisko G.L., Sanchez C. (2018). Nanocellulose, a Versatile Green Platform: From Biosources to Materials and Their Applications. Chem. Rev..

[17] Kargarzadeh H., Ioelovich M., Ahmad I., Thomas S., Dufresne A., Kargarzadeh H., Ahmad I., Thomas S., Dufresne A. (2017). Methods for Extraction of Nanocellulose from Various Sources. Handbook of Nanocellulose and Cellulose Nanocomposites.

[18] Trache D., Hussin M.H., Haafiz M.K.M., Thakur V.K. (2017). Recent Progress in Cellulose Nanocrystals: Sources and Production. Nanoscale.

[19] Nechyporchuk O., Belgacem M.N., Bras J. (2016). Production of Cellulose Nanofibrils: A Review of Recent Advances. Ind. Crops Prod..

[20] Abdul Khalil H.P.S., Davoudpour Y., Islam M.d.N., Mustapha A., Sudesh K., Dungani R., Jawaid M. (2014). Production and Modification of Nanofibrillated Cellulose Using Various Mechanical Processes: A Review. Carbohydr. Polym..

[21] Trache D., Donnot A., Khimeche K., Benelmir R., Brosse N. (2014). Physico-Chemical Properties and Thermal Stability of Microcrystalline Cellulose Isolated from Alfa Fibres. Carbohydr. Polym..

[22] Klemm D., Schumann D., Udhardt U., Marsch S. (2001). Bacterial Synthesized Cellulose—Artificial Blood Vessels for Microsurgery. Prog. Polym. Sci..

[23] Gorgieva S., Trček J. (2019). Bacterial Cellulose: Production, Modification and Perspectives in Biomedical Applications. Nanomaterials.

[24] Barja F. (2021). Bacterial Nanocellulose Production and Biomedical Applications. J. Biomed. Res..

[25] Habibi Y., Lucia L.A., Rojas O.J. (2010). Cellulose Nanocrystals: Chemistry, Self-Assembly, and Applications. Chem. Rev..

[26] Eichhorn S.J., Dufresne A., Aranguren M., Marcovich N.E., Capadona J.R., Rowan S.J., Weder C., Thielemans W., Roman M., Renneckar S. (2010). Review: Current International Research into Cellulose Nanofibres and Nanocomposites. J. Mater. Sci..

[27] Isogai A., Saito T., Fukuzumi H. (2011). TEMPO-Oxidized Cellulose Nanofibers. Nanoscale.

[28] Nogi M., Iwamoto S., Nakagaito A.N., Yano H. (2009). Optically Transparent Nanofiber Paper. Adv. Mater..

[29] Gama M., Dourado F., Bielecki S. (2016). Bacterial Nanocellulose: From Biotechnology to Bio-Economy.

[30] Lin W.-C., Lien C.-C., Yeh H.-J., Yu C.-M., Hsu S. (2013). Bacterial Cellulose and Bacterial Cellulose–Chitosan Membranes for Wound Dressing Applications. Carbohydr. Polym..

[31] Lavoine N., Desloges I., Dufresne A., Bras J. (2012). Microfibrillated Cellulose—Its Barrier Properties and Applications in Cellulosic Materials: A Review. Carbohydr. Polym..

[32] Kontturi E., Laaksonen P., Linder M.B., Nonappa, Gröschel A.H., Rojas O.J., Ikkala O. (2018). Advanced Materials through Assembly of Nanocelluloses. Adv. Mater..

[33] Missoum K., Belgacem M., Bras J. (2013). Nanofibrillated Cellulose Surface Modification: A Review. Materials.

[34] Eyley S., Thielemans W. (2014). Surface Modification of Cellulose Nanocrystals. Nanoscale.

[35] Sehaqui H., Salajková M., Zhou Q., Berglund L.A. (2010). Mechanical Performance Tailoring of Tough Ultra-High Porosity Foams Prepared from Cellulose I Nanofiber Suspensions. Soft Matter.

[36] Norizan M.N., Shazleen S.S., Alias A.H., Sabaruddin F.A., Asyraf M.R.M., Zainudin E.S., Abdullah N., Samsudin M.S., Kamarudin S.H., Norrrahim M.N.F. (2022). Nanocellulose-Based Nanocomposites for Sustainable Applications: A Review. Nanomaterials.

[37] Heinze T., Liebert T. (2001). Unconventional Methods in Cellulose Functionalization. Prog. Polym. Sci..

[38] Hokkanen S., Bhatnagar A., Sillanpää M. (2016). A Review on Modification Methods to Cellulose-Based Adsorbents to Improve Adsorption Capacity. Water Res..

[39] Si R., Wang D., Chen Y., Yu D., Ding Q., Li R., Wu C. (2021). Nanocelllulose Based Adsorbents for Heavy Metal Ions Removal. Preprint.

[40] Saito T., Isogai A. (2006). Introduction of Aldehyde Groups on Surfaces of Native Cellulose Fibers by TEMPO-Mediated Oxidation. Colloids Surf. A Physicochem. Eng. Asp..

[41] Saito T., Kimura S., Nishiyama Y., Isogai A. (2007). Cellulose Nanofibers Prepared by TEMPO-Mediated Oxidation of Native Cellulose. Biomacromolecules.

[42] Habibi Y., Dufresne A. (2008). Highly Filled Bionanocomposites from Functionalized Polysaccharide Nanocrystals. Biomacromolecules.

[43] Silva L.S., Ferreira F.J.L., Silva M.S., Citó A.M.G.L., Meneguin A.B., Sábio R.M., Barud H.S., Bezerra R.D.S., Osajima J.A., Silva Filho E.C. (2018). Potential of Amino-Functionalized Cellulose as an Alternative Sorbent Intended to Remove Anionic Dyes from Aqueous Solutions. Int. J. Biol. Macromol..

[44] Lavoine N., Bergström L. (2017). Nanocellulose-Based Foams and Aerogels: Processing, Properties, and Applications. J. Mater. Chem. A.

[45] Jiang F., Hsieh Y.-L. (2017). Cellulose Nanofibril Aerogels: Synergistic Improvement of Hydrophobicity, Strength, and Thermal Stability via Cross-Linking with Diisocyanate. ACS Appl. Mater. Interfaces.

[46] Iwamoto S., Nakagaito A.N., Yano H. (2007). Nano-Fibrillation of Pulp Fibers for the Processing of Transparent Nanocomposites. Appl. Phys. A.

[47] Henriksson M., Berglund L.A., Isaksson P., Lindström T., Nishino T. (2008). Cellulose Nanopaper Structures of High Toughness. Biomacromolecules.

[48] Frey M.W. (2008). Electrospinning Cellulose and Cellulose Derivatives. Polym. Rev..

[49] Toledo A.L.M.M., Da Silva T.N., Dos S., Vaucher A.C., Miranda A.H.V., Silva G.C.C., Vaz M.E.R., Silva L.V.D., Barradas T.N., Picciani P.H.S. (2021). Polymer Nanofibers for Biomedical Applications: Advances in Electrospinning. CAPS.

[50] Sehaqui H., Zhou Q., Berglund L.A. (2011). High-Porosity Aerogels of High Specific Surface Area Prepared from Nanofibrillated Cellulose (NFC). Compos. Sci. Technol..

[51] Nogi M., Yano H. (2008). Transparent Nanocomposites Based on Cellulose Produced by Bacteria Offer Potential Innovation in the Electronics Device Industry. Adv. Mater..

[52] Österberg M., Vartiainen J., Lucenius J., Hippi U., Seppälä J., Serimaa R., Laine J. (2013). A Fast Method to Produce Strong NFC Films as a Platform for Barrier and Functional Materials. ACS Appl. Mater. Interfaces.

[53] Hubbe M.A., Rojas O.J., Lucia L.A., Sain M. (2008). Cellulosic Nanocomposites: A Review. BioResources.

[54] Kalia S., Boufi S., Celli A., Kango S. (2014). Nanofibrillated Cellulose: Surface Modification and Potential Applications. Colloid. Polym. Sci..

[55] Hu L., Zheng G., Yao J., Liu N., Weil B., Eskilsson M., Karabulut E., Ruan Z., Fan S., Bloking J.T. (2013). Transparent and Conductive Paper from Nanocellulose Fibers. Energy Environ. Sci..

[56] Flynn C.N., Byrne C.P., Meenan B.J. (2013). Surface Modification of Cellulose via Atmospheric Pressure Plasma Processing in Air and Ammonia–Nitrogen Gas. Surf. Coat. Technol..

[57] Primc G., Mozetič M. (2024). Surface Modification of Polymers by Plasma Treatment for Appropriate Adhesion of Coatings. Materials.

[58] Jur J.S., Spagnola J.C., Lee K., Gong B., Peng Q., Parsons G.N. (2010). Temperature-Dependent Subsurface Growth during Atomic Layer Deposition on Polypropylene and Cellulose Fibers. Langmuir.

[59] Keskiväli L., Seppänen T., Porri P., Pääkkönen E., Ketoja J.A. (2023). Atomic Layer Deposited TiO_2_ on a Foam-Formed Cellulose Fibre Network—Effect on Hydrophobicity and Physical Properties. BioRes.

[60] Dufresne A. (2013). Nanocellulose: A New Ageless Bionanomaterial. Mater. Today.

[61] Gopakumar D.A., Pai A.R., Pottathara Y.B., Pasquini D., Carlos De Morais L., Luke M., Kalarikkal N., Grohens Y., Thomas S. (2018). Cellulose Nanofiber-Based Polyaniline Flexible Papers as Sustainable Microwave Absorbers in the X-Band. ACS Appl. Mater. Interfaces.

[62] Li D., Xia Y. (2004). Electrospinning of Nanofibers: Reinventing the Wheel?. Adv. Mater..

[63] Mishra R.K., Sabu A., Tiwari S.K. (2018). Materials Chemistry and the Futurist Eco-Friendly Applications of Nanocellulose: Status and Prospect. J. Saudi Chem. Soc..

[64] Lasrado D., Ahankari S., Kar K. (2020). Nanocellulose-based Polymer Composites for Energy Applications—A Review. J. Appl. Polym. Sci..

[65] Gibson L.J., Ashby M.F. (1997). Cellular Solids: Structure and Properties.

[66] Klemm D., Cranston E.D., Fischer D., Gama M., Kedzior S.A., Kralisch D., Kramer F., Kondo T., Lindström T., Nietzsche S. (2018). Nanocellulose as a Natural Source for Groundbreaking Applications in Materials Science: Today’s State. Mater. Today.

[67] Liang L., Huang C., Ragauskas A.J. (2017). Nanocellulose-Based Materials for Biomedical Applications. JSM Chem..

[68] Thakur V., Guleria A., Kumar S., Sharma S., Singh K. (2021). Recent Advances in Nanocellulose Processing, Functionalization and Applications: A Review. Mater. Adv..

[69] Bao Y., He J., Song K., Guo J., Zhou X., Liu S. (2022). Functionalization and Antibacterial Applications of Cellulose-Based Composite Hydrogels. Polymers.

[70] Li Y., Chen Y., Huang X., Jiang S., Wang G. (2021). Anisotropy-Functionalized Cellulose-Based Phase Change Materials with Reinforced Solar-Thermal Energy Conversion and Storage Capacity. Chem. Eng. J..

[71] Bethke K., Palantöken S., Andrei V., Roß M., Raghuwanshi V.S., Kettemann F., Greis K., Ingber T.T.K., Stückrath J.B., Valiyaveettil S. (2018). Functionalized Cellulose for Water Purification, Antimicrobial Applications, and Sensors. Adv. Funct. Mater..

[72] He W., Wu J., Xu J., Mosselhy D.A., Zheng Y., Yang S. (2021). Bacterial Cellulose: Functional Modification and Wound Healing Applications. Adv. Wound Care.

[73] Liang Y., He J., Guo B. (2021). Functional Hydrogels as Wound Dressing to Enhance Wound Healing. ACS Nano.

[74] Pinho E., Soares G. (2018). Functionalization of Cotton Cellulose for Improved Wound Healing. J. Mater. Chem. B.

[75] Pal S., Nisi R., Stoppa M., Licciulli A. (2017). Silver-Functionalized Bacterial Cellulose as Antibacterial Membrane for Wound-Healing Applications. ACS Omega.

[76] Alavi M., Nokhodchi A. (2020). An Overview on Antimicrobial and Wound Healing Properties of ZnO Nanobiofilms, Hydrogels, and Bionanocomposites Based on Cellulose, Chitosan, and Alginate Polymers. Carbohydr. Polym..

[77] Deng L., Wang B., Li W., Han Z., Chen S., Wang H. (2022). Bacterial Cellulose Reinforced Chitosan-Based Hydrogel with Highly Efficient Self-Healing and Enhanced Antibacterial Activity for Wound Healing. Int. J. Biol. Macromol..

[78] Basu A., Celma G., Strømme M., Ferraz N. (2018). In Vitro and in Vivo Evaluation of the Wound Healing Properties of Nanofibrillated Cellulose Hydrogels. ACS Appl. Bio Mater..

[79] El Allaoui B., Benzeid H., Zari N., Qaiss A.E.K., Bouhfid R. (2023). Functional Cellulose-Based Beads for Drug Delivery: Preparation, Functionalization, and Applications. J. Drug Deliv. Sci. Technol..

[80] Long W., Ouyang H., Zhou C., Wan W., Yu S., Qian K., Liu M., Zhang X., Feng Y., Wei Y. (2021). Simultaneous Surface Functionalization and Drug Loading: A Novel Method for Fabrication of Cellulose Nanocrystals-Based pH Responsive Drug Delivery System. Int. J. Biol. Macromol..

[81] Tang L., Lin F., Li T., Cai Z., Hong B., Huang B. (2018). Design and Synthesis of Functionalized Cellulose Nanocrystals-Based Drug Conjugates for Colon-Targeted Drug Delivery. Cellulose.

[82] Novotna K., Havelka P., Sopuch T., Kolarova K., Vosmanska V., Lisa V., Svorcik V., Bacakova L. (2013). Cellulose-Based Materials as Scaffolds for Tissue Engineering. Cellulose.

[83] Adhikari J., Dasgupta S., Barui A., Ghosh M., Saha P. (2023). Collagen Incorporated Functionalized Bacterial Cellulose Composite: A Macromolecular Approach for Successful Tissue Engineering Applications. Cellulose.

[84] Janmohammadi M., Nazemi Z., Salehi A.O.M., Seyfoori A., John J.V., Nourbakhsh M.S., Akbari M. (2023). Cellulose-Based Composite Scaffolds for Bone Tissue Engineering and Localized Drug Delivery. Bioact. Mater..

[85] Chainoglou E., Karagkiozaki V., Choli-Papadopoulou T., Mavromanolis C., Laskarakis A., Logothetidis S. (2016). Development of Biofunctionalized Cellulose Acetate Nanoscaffolds for Heart Valve Tissue Engineering. World J. Nano Sci. Eng..

[86] Akaya H., Lamnini S., Sehaqui H., Jacquemin J. (2025). Amine-Functionalized Cellulose as Promising Materials for Direct CO_2_ Capture: A Review. ACS Appl. Mater. Interfaces.

[87] Zafari R., Mendonça F.G., Tom Baker R., Fauteux-Lefebvre C. (2023). Efficient SO_2_ Capture Using an Amine-Functionalized, Nanocrystalline Cellulose-Based Adsorbent. Sep. Purif. Technol..

[88] Bidgoli H., Mortazavi Y., Khodadadi A.A. (2019). A Functionalized Nano-Structured Cellulosic Sorbent Aerogel for Oil Spill Cleanup: Synthesis and Characterization. J. Hazard. Mater..

[89] Gebald C., Wurzbacher J.A., Tingaut P., Steinfeld A. (2013). Stability of Amine-Functionalized Cellulose during Temperature-Vacuum-Swing Cycling for CO_2_ Capture from Air. Environ. Sci. Technol..

[90] Singh K., Sinha T.J.M., Srivastava S. (2015). Functionalized Nanocrystalline Cellulose: Smart Biosorbent for Decontamination of Arsenic. Int. J. Miner. Process..

[91] Ma L., Bi Z., Xue Y., Zhang W., Huang Q., Zhang L., Huang Y. (2020). Bacterial Cellulose: An Encouraging Eco-Friendly Nano-Candidate for Energy Storage and Energy Conversion. J. Mater. Chem. A.

[92] Lv P., Lu X., Wang L., Feng W. (2021). Nanocellulose-Based Functional Materials: From Chiral Photonics to Soft Actuator and Energy Storage. Adv. Funct. Mater..

[93] Chen W., Yu H., Lee S.-Y., Wei T., Li J., Fan Z. (2018). Nanocellulose: A Promising Nanomaterial for Advanced Electrochemical Energy Storage. Chem. Soc. Rev..

[94] Yang Y., Sun R., Wang X. (2017). Ag Nanowires Functionalized Cellulose Textiles for Supercapacitor and Photothermal Conversion. Mater. Lett..

[95] Xu X., Zhao G., Wang H., Li X., Feng X., Cheng B., Shi L., Kang W., Zhuang X., Yin Y. (2019). Bio-Inspired Amino-Acid-Functionalized Cellulose Whiskers Incorporated into Sulfonated Polysulfone for Proton Exchange Membrane. J. Power Sources.

[96] T. Vicente A., Araújo A., Mendes M.J., Nunes D., Oliveira M.J., Sanchez-Sobrado O., Ferreira M.P., Águas H., Fortunato E., Martins R. (2018). Multifunctional Cellulose-Paper for Light Harvesting and Smart Sensing Applications. J. Mater. Chem. C.

[97] Nawaz H., Zhang X., Chen S., You T., Xu F. (2021). Recent Studies on Cellulose-Based Fluorescent Smart Materials and Their Applications: A Comprehensive Review. Carbohydr. Polym..

[98] Zhao D., Zhu Y., Cheng W., Chen W., Wu Y., Yu H. (2021). Cellulose-Based Flexible Functional Materials for Emerging Intelligent Electronics. Adv. Mater..

[99] Dichiara A.B., Song A., Goodman S.M., He D., Bai J. (2017). Smart Papers Comprising Carbon Nanotubes and Cellulose Microfibers for Multifunctional Sensing Applications. J. Mater. Chem. A.

[100] Bishnoi P., Siwal S.S., Kumar V., Thakur V.K. (2024). Cellulose-based Smart Materials: Novel Synthesis Techniques, Properties, and Applications in Energy Storage and Conversion Devices. Electron.

[101] Jabir L., El-Hammi H., Mohammed N., Jilal I., El Idrissi A., Amhamdi H., Abou-Salama M., El Ouardi Y., El Barkany S., Laatikainen K. (2022). Cellulose Based pH-Sensitive Hydrogel for Highly Efficient Dye Removal in Water Treatment: Kinetic, Thermodynamic, Theoretical and Computational Studies. Cellulose.

[102] Geissler A., Bonaccurso E., Heim L.-O., Heinze T., Zhang K. (2014). Temperature-Responsive Thin Films from Cellulose Stearoyl Triester. J. Phys. Chem. C.

[103] Smyth M., Rader C., Bras J., Foster E.J. (2018). Characterization and Mechanical Properties of Ultraviolet Stimuli-responsive Functionalized Cellulose Nanocrystal Alginate Composites. J. Appl. Polym. Sci..

[104] Sekwele K.G., Tichapondwa S.M., Mhike W. (2024). Cellulose, Graphene and Graphene-Cellulose Composite Aerogels and Their Application in Water Treatment: A Review. Discov. Mater..

[105] Trache D., Thakur V.K., Boukherroub R. (2020). Cellulose Nanocrystals/Graphene Hybrids—A Promising New Class of Materials for Advanced Applications. Nanomaterials.

[106] Kamel S., Khattab T.A. (2020). Recent Advances in Cellulose-Based Biosensors for Medical Diagnosis. Biosensors.

[107] Ratajczak K., Stobiecka M. (2020). High-Performance Modified Cellulose Paper-Based Biosensors for Medical Diagnostics and Early Cancer Screening: A Concise Review. Carbohydr. Polym..

[108] Fahma F., Febiyanti I., Lisdayana N., Arnata I.W., Sartika D. (2021). Nanocellulose as a New Sustainable Material for Various Applications: A Review. Arch. Mater. Sci. Eng..

[109] Wong W. (2023). Paper-Based Biosensors for the Point-of-Care Detection of Human Serum Albumins—A Mini Review. ChemRxiv.

[110] Lu Z., Zhang H., Toivakka M., Xu C. (2024). Current Progress in Functionalization of Cellulose Nanofibers (CNFs) for Active Food Packaging. Int. J. Biol. Macromol..

[111] Han J., Chen M., Liu H., Zhang D., Shi Q., Xie X., Guo Y. (2024). Antimicrobial and Degradable All-Cellulose Composite for Functional and Sustainable Food Packaging. Ind. Crops Prod..

[112] Liu Y., Ahmed S., Sameen D.E., Wang Y., Lu R., Dai J., Li S., Qin W. (2021). A Review of Cellulose and Its Derivatives in Biopolymer-Based for Food Packaging Application. Trends Food Sci. Technol..

[113] Dandegaonkar G., Ahmed A., Sun L., Adak B., Mukhopadhyay S. (2022). Cellulose Based Flexible and Wearable Sensors for Health Monitoring. Mater. Adv..

[114] Rivadeneyra A., Marín-Sánchez A., Wicklein B., Salmerón J.F., Castillo E., Bobinger M., Salinas-Castillo A. (2021). Cellulose Nanofibers as Substrate for Flexible and Biodegradable Moisture Sensors. Compos. Sci. Technol..

[115] Fu Q., Cui C., Meng L., Hao S., Dai R., Yang J. (2021). Emerging Cellulose-Derived Materials: A Promising Platform for the Design of Flexible Wearable Sensors toward Health and Environment Monitoring. Mater. Chem. Front..

